# Comparison of low versus high (standard) intraabdominal pressure during laparoscopic colorectal surgery: systematic review and meta-analysis

**DOI:** 10.1007/s00384-024-04679-8

**Published:** 2024-07-10

**Authors:** Mohammed Hamid, Omar E. S. Mostafa, Ali Yasen Y. Mohamedahmed, Shafquat Zaman, Prajeesh Kumar, Peter Waterland, Akinfemi Akingboye

**Affiliations:** 1 Department of General Surgery, Wye Valley NHS Trust, Hereford County Hospital, Hereford, Herefordshire UK; 2 Department of General and Colorectal Surgery, Dudley Group NHS Foundation Trust, Russells Hall Hospital, Dudley, West Midlands UK; 3 Department of General Surgery, University Hospitals of Derby and Burton NHS Foundation Trust, Queen’s Hospital Burton, Burton on Trent, Staffordshire, UK; 4 College of Medical and Dental Sciences, School of Medicine, University of Birmingham, Edgbaston, Birmingham, UK

**Keywords:** Intraabdominal pressure, Laparoscopic colorectal surgery, Post-operative outcomes, Systematic review, Meta-analysis

## Abstract

**Background:**

To evaluate outcomes of low with high intraabdominal pressure during laparoscopic colorectal resection surgery.

**Methods:**

A systematic search of multiple electronic data sources was conducted, and all studies comparing low with high (standard) intraabdominal pressures were included. Our primary outcomes were post-operative ileus occurrence and return of bowel movement/flatus. The evaluated secondary outcomes included: total operative time, post-operative haemorrhage, anastomotic leak, pneumonia, surgical site infection, overall post-operative complications (categorised by Clavien-Dindo grading), and length of hospital stay. Revman 5.4 was used for data analysis.

**Results:**

Six randomised controlled trials (RCTs) and one observational study with a total of 771 patients (370 surgery at low intraabdominal pressure and 401 at high pressures) were included. There was no statistically significant difference in all the measured outcomes; post-operative ileus [OR 0.80; CI (0.42, 1.52), *P* = 0.50], time-to-pass flatus [OR -4.31; CI (-12.12, 3.50), *P* = 0.28], total operative time [OR 0.40; CI (-10.19, 11.00), *P* = 0.94], post-operative haemorrhage [OR 1.51; CI (0.41, 5.58, *P* = 0.53], anastomotic leak [OR 1.14; CI (0.26, 4.91), P = 0.86], pneumonia [OR 1.15; CI (0.22, 6.09), P = 0.87], SSI [OR 0.69; CI (0.19, 2.47), *P* = 0.57], overall post-operative complications [OR 0.82; CI (0.52, 1.30), *P* = 0.40], Clavien-Dindo grade ≥ 3 [OR 1.27; CI (0.59, 2.77), *P* = 0.54], and length of hospital stay [OR -0.68; CI (-1.61, 0.24), *P* = 0.15].

**Conclusion:**

Low intraabdominal pressure is safe and feasible approach to laparoscopic colorectal resection surgery with non-inferior outcomes to standard or high pressures. More robust and well-powered RCTs are needed to consolidate the potential benefits of low over high pressure intra-abdominal surgery.

## Introduction

It has become the gold standard, internationally, to approach elective cancer and benign colorectal resection surgery via laparoscopy. Compared to the open approach, benefits in favour of laparoscopy include the reduction of postoperative pain, consumption of analgesia, lower overall morbidity, shorter hospital stay, improved cosmesis, and patient satisfaction [1–5].

Significantly elevating abdominal pressures in laparoscopy is known to impact cardio-respiratory parameters. Therefore, attempts to further advance the laparoscopic approach has led to the investigation of intraabdominal pneumoperitoneum pressure effects on patient outcomes [6, 7].

European consensus guidelines recommend the use of the lowest possible pressure that maintains sufficient visibility to safely perform the procedure [8]. The recent “low-pressure versus standard-pressure laparoscopic colorectal surgery” (PAROS) randomised controlled trial (RCT), compared pressures of 5–7 mmHg (low-experimental) to 12–15 mmHg (high-standard). They demonstrated improved post-operative recovery (including reduced postoperative pain and analgesia utilisation) and shorter hospital stay in favour of low pressures [9]. Studies comparing low versus high pressures in laparoscopic cholecystectomies have also recorded similar results [10–12]. Conversely, other studies have associated low pressures with adverse impact on the quality, safety and timing of surgery [10–14].

We performed a systematic review and meta-analysis of the available evidence, to evaluate outcomes of low with high intraabdominal pressure during laparoscopic colorectal resections, to elucidate conclusions and recommendations for its use in routine clinical practice.

## Methods

The Cochrane Handbook for Systematic Reviews of Interventions and Preferred Reporting Items for Systematic Reviews and Meta-Analyses (PRISMA) guidelines were used in the design of this review [15, 16], and it was registered at the International Prospective Register of Systematic Reviews (registration number: CRD42024531307 available at: https://www.crd.york.ac.uk/prospero).

The included papers were based on the following PICO (Population, Intervention, Comparator, Outcomes) format:

***Population:*** All patients undergoing laparoscopic colorectal resections.

***Intervention:*** Low intraabdominal pneumoperitoneum pressures.

***Comparator:*** Standard or high intraabdominal pneumoperitoneum pressures.

***Outcomes:*** Post-operative ileus occurrence and first return of bowel movement/flatus were our primary outcomes. Investigated secondary outcomes were as follows: total operative time, post-operative haemorrhage, anastomotic leak rate, pneumonia, surgical site infection (SSI), overall post-operative complications (categorised by Clavien-Dindo grading), and length of hospital stay. Moreover, post-operative pain scores and analgesia requirement were also reviewed.

## Study design

A systematic review and meta-analysis of comparative studies was conducted. We excluded the following: case-series, case reports, letters to the editor, non-comparative single-arm reports, and data presented as a conference abstract and published online.

## Search strategy

The following on-line electronic databases and clinical trial registers were searched: PubMed, MEDLINE, ScienceDirect, Embase, Scopus, clinical trials.gov, and Cochrane Central Register of Controlled Trials (CENTRAL), up to and including 15/04/2024. Language restrictions and filters were applied. In addition, a manual search of the reference lists and bibliographies cited in previous reviews was performed to identify any missed studies.

We used a combination of the following terms to conduct our search: *"abdominal pressure” OR "intraabdominal pressure “OR "pneumoperitoneum" AND "colorectal” OR “colorectal surgery” AND "laparoscopic" OR “laparoscopy”.* Two authors performed the database search and reviewed the extracted articles independently.

## Eligibility and study selection criteria

**Fig. 1:**
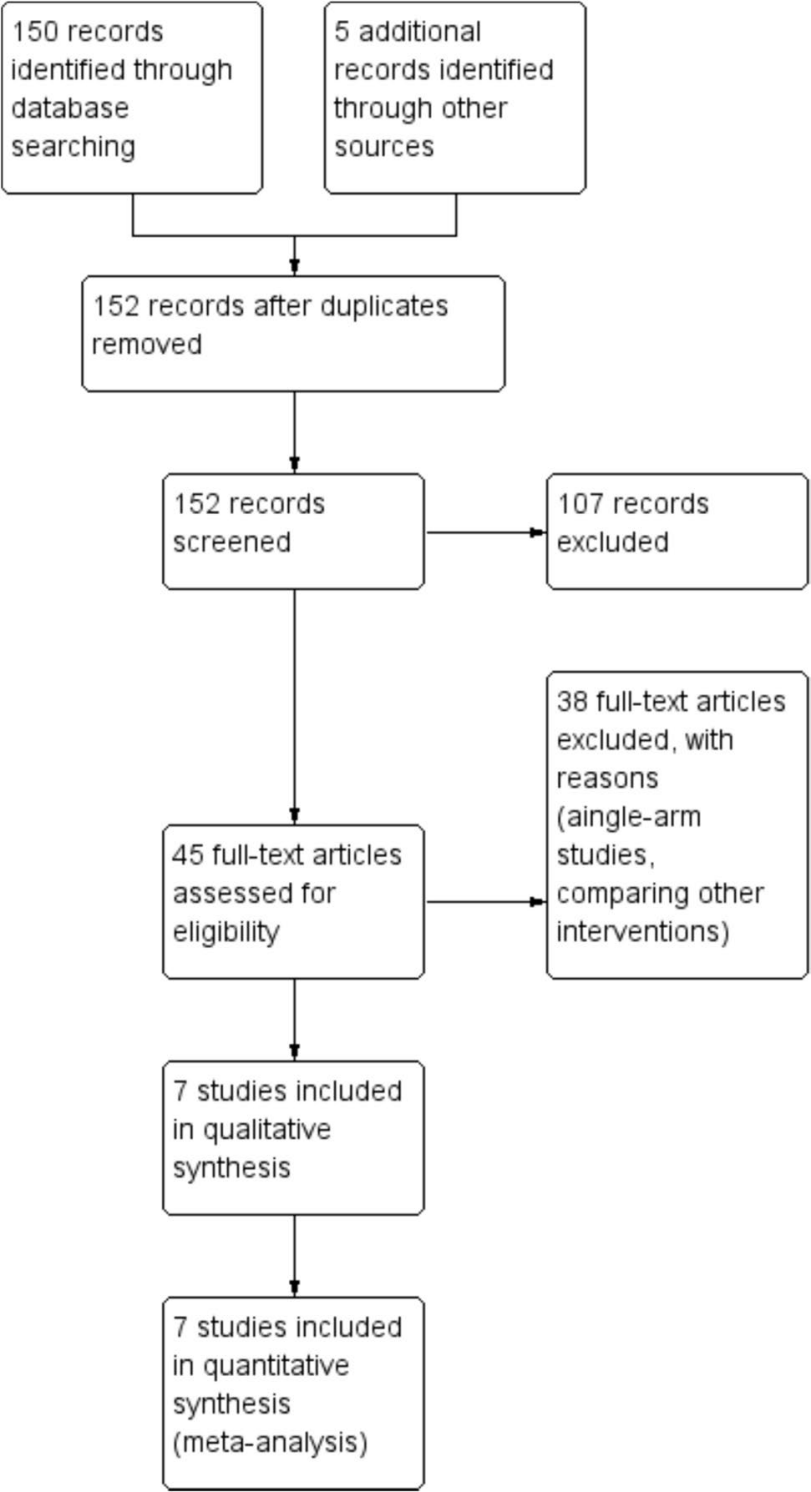
PRISMA flow chart

Articles included in this analysis were based on the PICO framework outlined above. We excluded duplicate studies and literature comparing other techniques such as the gas (e.g. CO_2_ vs. N_2_O) used to establish pneumoperitoneum. The titles and abstracts that were selected, were evaluated for relevance independently by two authors. Studies were marked as either included, excluded, or requiring further evaluation. The full-text of the articles matching the inclusion criteria were sourced (Fig. 1).

Any disagreement in the selection of the studies were resolved by discussion with a third reviewer. If any further discrepancy persisted, counsel was taken from the complete authorship panel to reach a consensus.

## Data extraction and collection

An electronic spreadsheet (as per the Cochrane recommendations) for data extraction was formulated in Microsoft Excel. Following pilot-testing with randomly selected articles, the spreadsheet was modified and adjusted accordingly to create a final version. Two reviewers independently extracted data from the hand-selected studies. Data extracted included:

- Study-related data: authorship, year of publication, country in which the study was performed, study design, number of patients in each arm, and inclusion/exclusion criteria (Table 1).
- Baseline demographic and clinical information of the study population (Table 2).
- Primary and secondary outcomes.

**Table 1:** Characteristics of included studies

**Study**	**Country**	**Type of Study**	**Population**	**Pressure** **Measurements** **(mmHg)**	**Planned Primary Outcomes**	**Inclusion and Exclusion criteria**	**NMB level**	**Duration of follow-up**
Cai et al 2015 [17]	China	RCT	**LP** 22 **HP** 44	10, 12, 15	Time to flatus and bowel opening	**Inclusion criteria:** Age 40 to 80 years; ASA I–II; a biopsy proven histological diagnosis of colorectal carcinoma; no clinical evidence of metastasis; undergoing laparoscopic colorectal surgery **Exclusion criteria:** Contraindication of laparoscopic surgery (e.g. extensive intra-abdominal adhesion); emergency procedure; evidence of bowel ileus/obstruction before surgery; unresectable mass; a planned stoma (e.g. abdominoperineal resection of rectal carcinoma, protective ileal stoma); an unexpected stoma; conversion to open surgery; short-term re-operation; postoperative opioid analgesic usage; or persistent uncorrected severe fluid and electrolyte imbalance (e.g. hypokalaemia, hypomagnesemia)	N/A	7–14 days
Cho et al 2017 [18]	Korea	RCT	**LP** 44 **HP** 87	8 *vs.* 12	Cardiac Index	**Inclusion criteria:** Age 20 to 80 years; AS I–II; undergoing elective laparoscopic colorectal surgery **Exclusion criteria:** Patients with allergies to anaesthetic drugs; neuromuscular dysfunctions; severe cardiovascular or respiratory diseases; irregular cardiac rhythms; BMI > 35 kg/m^2^	**LP:** Deep-block (post-tetanic count 1–2) **HP:** Deep-block (*n* = 44) + moderate-block (TOF 1–2) (*n* = 43)	N/A Early post operative period not specified
Diaz-Cambronero et al 2020 [19]	Spain	RCT	**LP** 85 **HP** 81	Lowest possible *vs.* 12	Postoperative Quality of Recovery Scale	**Inclusion criteria:** Aged over 18 years; ASA below IV; patients scheduled for laparoscopic colorectal surgery **Exclusion criteria:** Absence of written informed consent; emergency or unplanned surgery; pregnancy or breastfeeding; immunological or neuromuscular diseases; advanced stage of cardiopulmonary, renal or hepatic disease; cognitive deficits; and allergy or contraindications to rocuronium or sugammadex	**LP:** Deep-block TOF 0 and post-tetanic count 1–5 **HP:** Moderate-block (TOF 2–4)	N/A Early post operative period not specified
Grieco et al 2021 [20]	Italy	Cohort study	**LP** 53 **HP** 21	12 *vs.* 15	Postoperative complications	**Inclusion criteria:** Adults with mid and low-level rectal cancer undergoing elective double-team TaTME with colorectal anastomosis and diverting ileostomy **Exclusion criteria:** Conversion to open surgery; ASA class IV; benign disease; synchronous tumours; procedures with terminal stoma/no colorectal anastomosis; and multi-visceral resection	N/A	30-days
Celaier et al 2021 [9]	France	RCT	**LP** 62 **HP** 65	7 *vs.* 12	Length of Stay	**Inclusion criteria:** Age over 18 years; undergoing a right or left colectomy for a malignant or benign pathology; planned laparoscopic procedure **Exclusion criteria:** Non-laparoscopic procedure; transverse or total colectomy; other procedure performed simultaneously with colonic surgery (except appendectomy or liver biopsy); emergency surgery; surgery for pelvic sepsis; procedure including de-functioning stoma; pregnant women; likely to be or breastfeeding; any patient incapable of providing informed consent; and those unable to commit to the medical follow-up of the study for geographical, social or psychological reasons	**LP + HP:** Deep-block post-tetanic count 3–5	30-days
Albers et al 2022 [21]	Netherlands	RCT	**LP** 89 **HP** 89	8 *vs.* 12	Quality of Recovery	**Inclusion criteria:** Age over 18 years; scheduled for laparoscopic colorectal surgery with a primary anastomosis; obtained informed consent **Exclusion criteria:** Insufficient control of the Dutch language to read the patient information and to fill out the questionnaires; primary colostomy; neo-adjuvant chemotherapy; chronic use of analgesics or psychotropic drugs; use of NSAIDs shorter than 5 days before surgery; known or suspected allergy to rocuronium of Sugammadex; neuromuscular disease; indication for rapid sequence induction; severe liver- or renal disease (creatinine clearance < 30 ml/min); BMI > 35 kg/m^2^; deficiency of vitamin K dependent clotting factors or coagulopathy	**LP:** deep-block post tetanic count 1–2 **HP:** Moderate-block TOF 1–2	30-day outcomes; 3-months HRQOL and MPQ
Arnal et al 2023 [22]	Spain	RCT	**LP** 15 **HP** 14	8 *vs.* 12	Liver Perfusion	**Inclusion criteria:** Age over 18 years; ASA I–III; previously signed informed consent; undergoing laparoscopic surgery **Exclusion criteria:** ASA over IV; pregnancy; advanced liver, kidney or cardiopulmonary disease	N/A	N/A Early post operative period not specified

*RCT* randomised controlled trial, *NMB* Neuromuscular block level, *LP* Low pressure, *HP* High pressure, *ASA* American Society of Anaesthesiologists grade, *BMI* Body Mass Index, *TaTME* Transanal total mesorectal excision, *TOF* train-of-four count, *HRQOL* Health-related quality of life, *MPQ* McGill Pain Questionnaire, *N/A* not available

**Table 2:** Patient demographics in the included studies

Study	Age mean ± SD / median (range)	Gender M: F	BMI mean ± SD / median (range)	Comorbidities (%)		Surgery Type (%)					
				ASA	Respiratory disease (%)	Oncologic resection	***Right hemi-colectomy	Left hemi-colectomy	Sigmoid colectomy	Anterior resection	Sub / Total colectomy
Cai et al 2015 [17]	LP: 61.9 ± 8.7 HP: 64.5 ± 9.1	LP: 14:8 HP: 27:17	LP: N/A HP: N/A	LP: N/A HP: N/A	LP: N/A HP: N/A	LP: 100 HP: 100	LP: 9.1 HP: 36.4	LP: 9.1 HP: 4.5	LP: 0 HP: 6.8	LP: 68.2 HP: 45.5	LP: 13.6 HP: 6.8
*Cho et al 2017 [18]	LP: 62 (11) HP: 66 (12)	LP: 16:28 HP: 35:52	LP: 22.9 (2.6) HP: 23.3 (2.6)	LP: I (45.5), II (54.5) HP: I (42.5), II (57.5)	LP: 0 HP: 0	LP: N/A HP: N/A	LP: N/A HP: N/A	LP: N/A HP: N/A	LP: 36.4 HP: 48.4	LP: 25.0 HP: 23.0	LP: N/A HP: N/A
**Diaz-Cambronero et al. 2020 [19]	LP: 68 (58–74) HP: 67 (59–77)	LP: 58:27 HP: 45:36	LP: 27.0 (24.2–29.9) HP: 26.6 (23.8–29.0)	LP: I (15), II (55), III (29) HP: I (15), II (60), III (25)	LP: 11 HP: 7	LP: 93 HP:86	LP: 35 HP: 38	LP: 5 HP: 1	LP: 20 HP: 20	LP: 28 HP: 26	LP: 1 HP: 1
Grieco et al 2021 [20]	LP: 68.7 ± 10.6 HP: 72.4 ± 9.6	LP: 37:16 HP: 12:9	LP: 26.5 ± 4.1 HP: 23.7 ± 3.6	LP: I (3.8), II (86.8), III (9.4) HP: I (9.5), II (85.7), III (4.8)	LP: N/A HP: N/A	LP: 100 HP: 100	LP: 0 HP: 0	LP: 0 HP: 0	LP: 0 HP: 0	LP: 100 HP: 100	LP: 0 HP: 0
Celaier et al 2021 [9]	LP: 65 (20–87) HP: 67 (22–93)	LP: 55:45 HP: 46:55	LP: 24.3 (16.3–38.1) HP: 23.0 (16.2–43.3)	LP: I (27), II (61), III (11) HP: I (34), II (54), III (12)	LP: N/A HP: N/A	LP: 60 HP: 59	LP: 36 HP: 45	LP: 26 HP: 25	LP: 39 HP: 31	LP: 0 HP: 0	LP: 0 HP: 0
Albers et al 2022 [21]	LP: 68.5 ± 9.5 HP: 68.9 ± 9.2	LP: 57:32 HP: 57:32	LP: 26.2 ± 4.0 HP: 27.3 ± 4.8	LP: I (21), II (63), III (16) HP: I (25), II (54), III (21)	LP: N/A HP: N/A	LP: 90 HP: 88	LP: 40 HP: 35	LP: 9 HP: 7	LP: 28 HP: 38	LP: 21 HP: 18	LP: 1 HP: 2
Arnal et al 2023 [22]	LP: 64 (58–69) HP: 71 (63–77)	LP: 15:0 HP: 9:5	LP: 27.1 (3.7) HP: 26.5 (3.6)	LP: I (13.3), II (60.0), III (26.7) HP: I (7.1), II (71.4), III (21.4)	LP: 0 HP: 0	LP: N/A HP: N/A	LP: 20.0 HP: 42.9	LP: 6.7 HP: 0.0	LP: 20.0 HP: 14.3	LP: 46.7 HP: 28.6	LP: 0.0 HP: 7.1

*Documented all hemicolectomies using on figure, LP: 38.6%; HP: 28.8%

** Other surgeries (Ileocecal resection, perineal amputation, segmental resection), LP: 11%; HP 14%

***Right hemicolectomy category includes ileocecal resections in studies separating both with clear documentation

*SD* standard deviation, *LP* Low pressure, *HP* High pressure, *BMI* Body Mass Index, *ASA* American Society of Anaesthesiologists grade, *N/A* not available

The authorship panel were consulted for the resolution on any disagreements arising during this process.

## Assessment of bias

The Newcastle–Ottawa Scale (NOS) was used to assess the risk of bias for the included observational study (Table 3) [23]. This quality assessment tool is based on a ‘star system’ and judges on three broad perspectives: selection of the study groups; the comparability of these groups; and the ascertainment of either the exposure (case–control studies) or outcome (cohort studies) of interest.

**Table 3:** Risk of bias assessment for the observational study using the Newcastle–Ottawa Scale

Study	Representativeness of the exposed cohort	Selection of the non-exposed cohort	Ascertainment of exposure	Demonstration that outcome of interest was not present at start of the study	Comparability of cohorts based on the design or analysis controlled for confounders	Assessment of outcome	Was follow-up long enough for outcomes to occur	Adequacy of follow-up of cohorts	Total
Grieco et al. 2021 [20]	*	*	*	*		*	*	*	7

* = 1 point; ** = 2 points

Studies were categorised as low risk when the total NOS score was 9, medium risk with scores of 7 or 8, and high risk of bias for scores ≤ 6, respectively. Any variance at this stage between the reviewers was reconciled through involvement of the authorship panel.

**Fig. 2:**
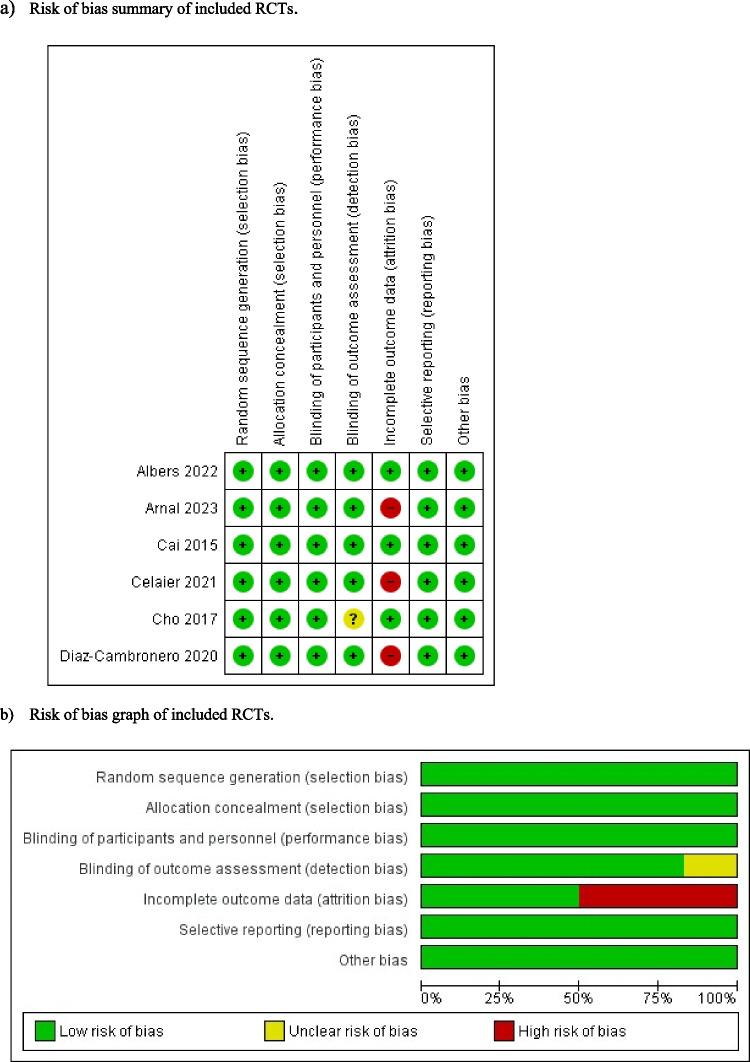
Risk of bias assessment of included randomised controlled trials (RCTs)

For RCTs, the risk of bias was assessed through the Cochrane risk of bias tool (Fig. 2) [24]. The following categories were classified as high, low, or unclear: random sequence generation, allocation concealment, blinding of participants and personnel, blinding of outcome assessment, incomplete outcome data, selective reporting, and other sources of bias.

## Statistical analysis

The statistical analysis in this review was performed using Review Manager (RevMan) 5.4 (Nordic Cochrane Centre, Cochrane Collaboration). The odds ratio (OR) and mean difference (MD) were analysed with their associated 95% confidence intervals (CI) using the Mantel–Haenszel method for dichotomous outcomes and continuous outcomes, respectively. If mean values were not available for continuous outcomes, then the median and interquartile range (IQR) were extracted. The equation described by Hozo et al. was used to convert this data to estimate the mean and standard deviation (SD) [25]. A random-effects model was used in all analyses.

The statistical significance threshold was set at *P* < 0.05. The I^2^ statistic using Cochrane Q test (χ^2^) was used to quantify between-study heterogeneity. High values of χ^2^ and I^2^ signify increasing levels of heterogeneity, with an I^2^ value of 0–50% representing low heterogeneity and > 50% considerable high heterogeneity. To check for possible causes of heterogeneity and evaluate the robustness of the results, sensitivity analysis was performed using the Risk Ratio (RR) and Risk Difference (RD).

## Results

Our literature search yielded a total of 155 articles. After removing duplicates and excluding irrelevant studies, 45 were potentially eligible to be included in this review. The full-text of these articles were retrieved and meticulously reviewed. This process resulted in seven articles identified as being eligible for inclusion in our data synthesis [9, 17–22]. The PRISMA flow chart is shown in Fig. 1.

A total of 771 patients were analysed, divided between low intraabdominal pressure (*n* = 370) and high or standard pressures (*n* = 401). Six of the included studies were RCTs (*n* = 697) [9, 17–19, 21, 22], and one was observational [20].

Tables 1 and 2 show the characteristics of the included studies and baseline demographic information.

## Risk of bias assessment

Table 3 highlights the outcomes of the methodological quality assessment, based on the NOS for the single observational study.

Risk of bias assessment for the included RCTs is shown in Fig. 2.

## Primary outcomes

### Paralytic ileus

**Fig. 3:**
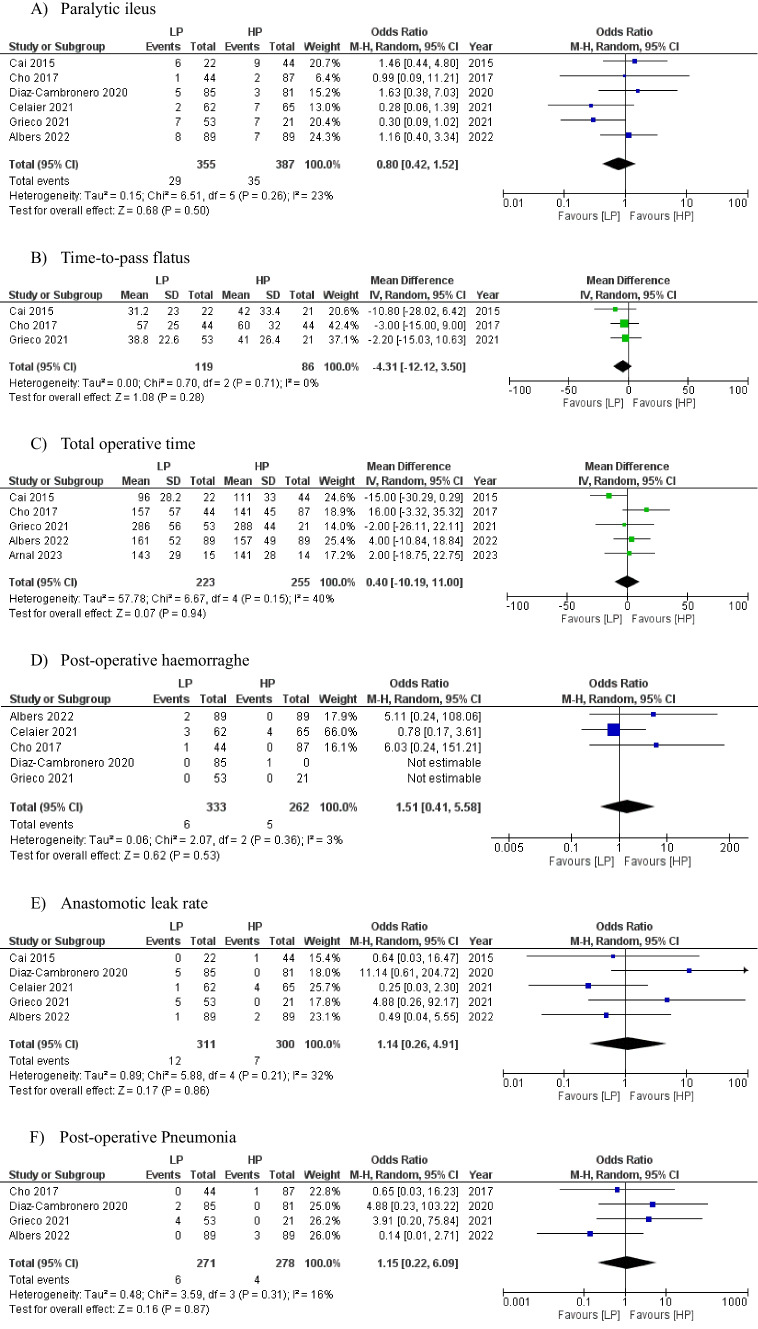

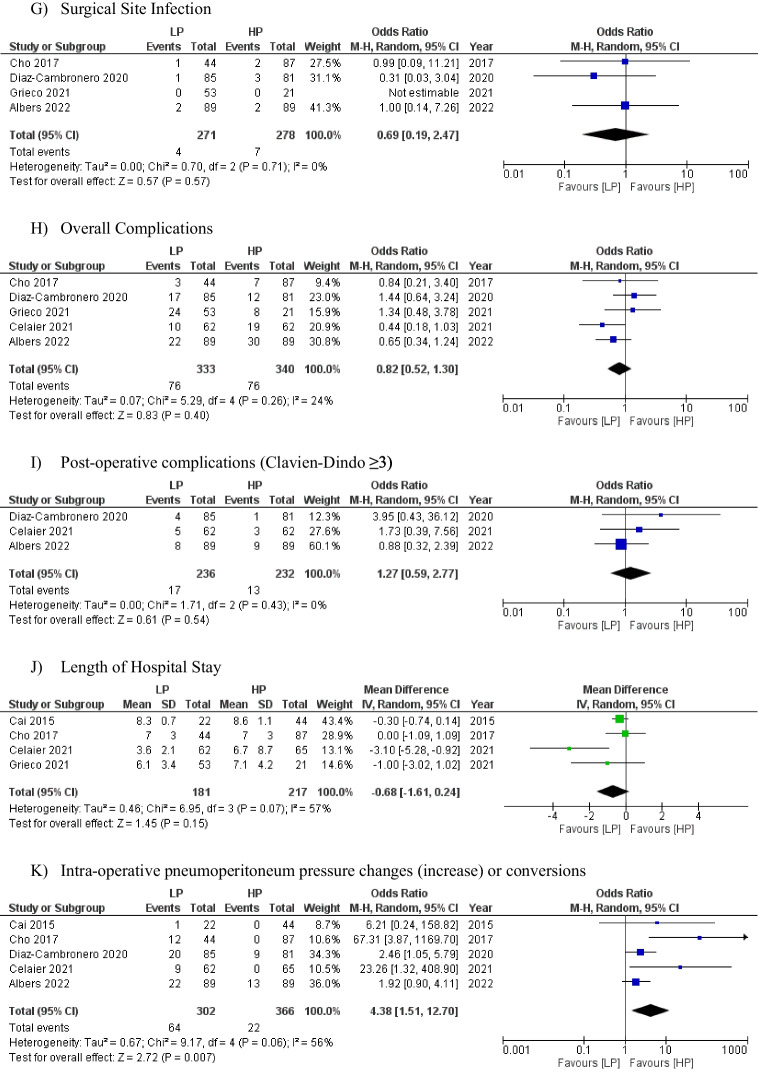
Forest plots of comparison of (**a**) paralytic ileus, (**b**) time-to-pass flatus, (**c**) total operative time, (**d**) post-operative haemorraghe, (**e**) anastomotic leak rate, (**f**) post-operative pneumonia, (**g**) surgical site infection, (**h**) overall complications, (**i**) post-operative complications (Clavien-Dindo ≥ 3), (**j**) length of hospital stay, and (**k**) intra-operative pneumoperitoneum pressure changes (increase) or conversions. The solid squares denote the mean difference or odds ratio. The horizontal lines represent the 95% confidence intervals (CIs), and the diamond denotes the pooled effect size. LP, low pressure; HP, high pressure; M–H, Mantel–Haenszel test

Six studies (*n* = 742) [9, 17–21] reported on paralytic ileus as a post-operative complication and the pooled analysis revealed a non-significantly lower rate in the low-pressure group (7.8% vs. 8.7%), [OR: 0.80 (0.42, 1.52) 95% CI, *P* = 0.50]. The level of heterogeneity was low among the included studies (I^2^ = 23%, *P* = 0.26) (Fig. 3-a).

### Time-to-pass flatus

Three studies documented time to pass flatus or first bowel movement (*n* = 271) [17, 18, 20]. It took a shorter, non-statistically significant, period for patients undergoing low-pressure surgery to pass flatus (42.3 vs. 47.7 h), [OR: -4.31 (-12.12, 3.50) 95% CI, *P* = 0.28]. The level of heterogeneity was low among the included studies (I^2^ = 0%, *P* = 0.71) (Fig. 3-b).

## Secondary outcomes

### Total operative time

Duration of surgery was reported in five studies (*n* = 478) [17, 18, 20–22]. The low-pressure group was associated with a longer (non-significant) total operative time (mean:168.6 min) in comparison to the high-pressures group (mean:167.6 min) [OR: 0.40 (-10.19, 11.00) 95% CI, *P* = 0.94]. The level of heterogeneity was low among the included studies (I^2^ = 40%, *P* = 0.15) (Fig. 3-c).

### Post-operative haemorrhage

Bleeding in the postoperative period was reported as an outcome in five studies [9, 18–21], (*n* = 676). It was non-significantly higher in the low-pressure group (1.6%) compared with the high-pressure group (1.2%), [OR: 1.51 (0.41, 5.58) 95% CI, *P* = 0.53]. The level of heterogeneity was low among the included studies (I^2^ = 3%, *P* = 0.36) (Fig. 3-d).

### Anastomotic leak rate

The overall rate of anastomotic leak was 2.5% across the two groups (five studies, *n* = 611) [9, 17, 19–21]. A higher (non-significant) rate was observed in the low-pressure group (3.2%) vs. the high-pressure group (1.7%) [OR: 1.14 (0.26, 4.91) 95% CI, *P* = 0.86]. The level of heterogeneity was low among the included studies (I^2^ = 32%, *P* = 0.21) (Fig. 3-e).

### Pneumonia

Chest infection in the post-operative period was reported as an outcome in four studies [18–21], (*n* = 549) and was non-significantly higher in the low-pressure group compared with the high-pressure group (1.6% vs. 1.0%), [OR: 1.15 (0.22, 6.09) 95% CI, *P* = 0.53]. The level of heterogeneity was low among the included studies (I^2^ = 16%, *P* = 0.31) (Fig. 3-f).

### Surgical site infection

Four studies recorded the rate of surgical site infection (*n* = 549) [18–21]. A lower non-significant rate was observed in the low-pressure group (1.1%) vs. the high-pressure group (1.7%), [OR: 0.69 (0.19, 2.47) 95% CI, *P* = 0.57]. The level of heterogeneity was low among the included studies (I^2^ = 0%, *P* = 0.71) (Fig. 3-g).

### Overall complications

Five studies documented on the overall number of post-operative complications (*n* = 671) [9, 18–21]. There was an overall higher, non-significant, post-operative complication rate in the low-pressure group (20.5% vs. 19.0%), [OR: 0.82 (0.52, 1.30) 95% CI, *p* = 0.40]. The level of heterogeneity was low among the included studies (I^2^ = 24%, *P* = 0.26) (Fig. 3-h).

### Post-operative complications (Clavien-Dindo ≥ 3)

Three studies [9, 19, 21] reported on post-operative complications with Clavien-Dindo grade ≥ 3, with a total number of 471 patients. There was a higher non-significant probability of increased complications (CD ≥ 3) in patients undergoing low intraabdominal pneumoperitoneum pressures during colorectal surgery [OR: 1.27 (0.59, 2.77) 95% CI, *P* = 0.54]. A low level of heterogeneity was observed between the included studies (I^2^ = 0%, *P* = 0.43) (Fig. 3-i).

### Length of hospital stay

Four studies [9, 17, 18, 20] compared length of stay (LOS) between the two groups with a total of 398 patients. The low-pressure group showed a reduced (non-significant) LOS (mean: 6.3 days) compared with the high-pressure group (mean: 7.4 days), [OR: -0.68 (-1.61, 0.24) 95% CI, *P* = 0.15]. The level of heterogeneity was high among the included studies (I^2^ = 57%, *P* = 0.07) (Fig. 3-j).

### Intra-operative pressure changes

Five studies documented the number of cases requiring a pressure increase or change (*n* = 668) [9, 17–19, 21]. As expected, there was a higher rate of intra-operative pressure change during low-pressure surgery (17.3%) compared to procedures already commencing at high or standard pressures (5.5%), [OR: 4.38 (1.51, 12.70) 95% CI, *P* = 0.007]. The level of heterogeneity was high among the included studies (I^2^ = 56%, *P* = 0.06) (Fig. 3-k).

Cho et al. [18], does not specify the pressure changes undertaken, but reported 12 low-pressure cases requiring an increase to higher pressures as the surgeon’s views were unacceptable. Diaz-Cambronero et al. [19], recorded more frequent requests for an increase in pressure in the low-pressure group (24% vs. 9%), and described this as mainly occurring during the pelvic phase of the procedure. Celarier et al. [9], documented poor exposure in 23% of the low-pressure cases, and this was strongly linked to obesity in 81% of those reported. Albers et al. [21], recorded 25% (low-pressure group) vs. 15% cases of intra-operative pressure changes, but reported no significant differences in the quality of the surgical field between the two groups.

### Post-operative pain

Four RCTs documented on post-operative pain and analgesia consumption, although each study utilised differing parameters to score pain and time-intervals to record this data. All except one study showed a significant reduction in post-operative pain after low-pressure surgery relative to higher pressures. Celaier et al. [9], recorded pain at 2, 8 and 24 hrs post operatively and concluded significantly lower reported pain (Visual Analogue Scale (VAS) ≤ 3, not clearly defined) at 2-hrs (76% vs 59%, P= 0038) and 8-hrs (87% vs 72%, *P* = 0.039) for low-pressure surgery, with the benefit tapering off after 24-hrs (77% vs 72%, *P* = 0.507). This study also found a significant reduction in both level 2 and level 3 WHO analgesia consumption amongst the low-pressure surgery group, (73% vs. 88%, *P* = 0.032) and (10% vs. 23%, *P* = 0.042), respectively.

Albers et al. [21] recorded pain using the QoR-40 score at daily intervals, with significantly lower reported pain at rest after the operation (4.7 vs. 5.8, *P* = 0.004), post-operative day (POD) 1 (2.7 vs. 3.3, *P* = 0.016) and day 3 (1.5 vs. 2.2, *P* = 0.015). Opioid analgesic consumption was however non-significant between the groups at each interval.

Diaz-Cambronero et al. [19], utilised the PQRS score at 15 min, 40 min, POD1, and POD3. The PQRS scored significantly in favour of low individualised pneumoperitoneum pressures with regards to nociceptive recovery, (OR: 0.47 (0.22, 0.99) 95% CI, *P* = 0.047; RR: 0.29 (0.16, 0.78) 95% CI, *P* = 0.023).

Cho et al. [18], recorded post-operative pain (at least 30 min, not clearly defined) using the VAS score (0, no pain, to 10, worst pain imaginable). No significant difference between the low- and standard-pressure groups was identified (5.5 vs. 5.35, *P* = 0.241).

### Sensitivity analysis

The direction of the pooled effect size remained unchanged when RR or RD was calculated for dichotomous variables. Furthermore, ‘leave-one-out’ analysis did not demonstrate any important discrepancies with the original analysis.

## Discussion

Laparoscopy has become a favourable approach for oncological and benign colorectal surgery, both in the elective and emergency settings, due to its notable peri-operative benefits. A minimum pneumoperitoneum pressure is vital for intra-operative visualisation; however, the effects of carbon dioxide (CO_2_) insufflation and the pressure at which this is sustained have been reported to impact cellular and metabolic functions of the body and peritoneum [8, 21, 26]. Although recent systematic reviews have provided comparative reports on low versus high (or standard) pneumoperitoneum pressure use in several laparoscopic procedures [8, 27, 28], to date, there is no level one evidence specific to the field of colorectal surgery. Accounting for the limitations, this systematic review and meta-analysis of six randomised controlled trials and one retrospective cohort study, substantiates the notion that low pneumoperitoneum pressure is a safe, non-inferior option in laparoscopic colorectal surgery.

The current literature-base reports various results, both in favour and against low-pressure laparoscopy, with support rising for its recommendation. In a meta-analysis of 85 studies, combining several subspecialty laparoscopic procedures, Reijnders-Boerboom et al. reported statistically significant lower incidence of post-operative complications including nausea and vomiting, reduced pain scores and length of hospital stay after low-pressure surgery [29]. Although strong analytical power is offered by this large collection, there were a significant number of poor-quality studies covered, including a considerable number of gynaecological, endocrine, and hepatobiliary procedures.

Ortenzi et al. conducted a meta-analysis on laparoscopic cholecystectomies, and found no statistical significance in post-operative complications, operative duration and post-operative stay for patients undergoing standard- versus low-pressure surgery [28].

Hua et al. also reported similar findings for post-operative complications in 1,263 patients undergoing laparoscopic cholecystectomy, with a slightly lower post-operative hospital stay in the low-pressure group [10]. In both reviews, post-operative pain and analgesia requirements were significantly reduced in the low-pressure groups compared with standard pressure, which has implications on patient comfort and cost-savings [10, 28].

The caveat to using lower pressures during laparoscopy centres around the quality of the surgical field which may infringe upon the safety of the procedure, and notably, Hue et al. observed that the pressure was increased six times more often in the lower pressure groups [10]. It is worthy to note that increasing the pressure in laparoscopic surgery, to ensure adequate visibility, is a sensible and safe decision available to surgeons, and should not be seen as a failure or complication of low-pressure laparoscopy. Given that colorectal surgery for malignancy and inflammatory conditions, present different technical challenges, approach, and demand to other surgeries, the above reported findings cannot be fully extrapolated.

Our review has found a lack of statistical significance between low- and standard-pressure use in laparoscopic colorectal surgery for all the measured outcomes, highlighting the safe use of lower pressures.

Unfortunately, we were unable to meta-analyse post-operative pain scores and analgesia requirements due to the significant heterogeneity in reporting methods between the studies. However, within the individual studies reporting on pain and analgesia consumption, lower pressures appear to significantly reduce nociception, particularly in the early stages of recovery. As expected, we observed a significant number of cases within the low-pressure groups undergoing conversion to higher pressures due to poor intra-operative visibility. Cases involving intraoperative pressure increases were excluded from within the studies, and it is worthwhile noting that the as-treated outcomes demonstrated a higher degree of significance in comparison to the intention-to-treat analysis, as highlighted in the PAROS trial [9].

Creating the optimal intra-abdominal volume, to the surgeon's satisfaction, can be achieved in a multitude of ways. Patient positioning, pre-stretching of the abdominal wall, intra-operative ventilation using low tidal volumes, and the use of deep neuromuscular blockade have all been shown to improve surgical conditions to varying degrees, allowing the use of lower intra-operative pressures, as well as directly influencing patient-outcomes [30–34]. A multifaceted cause and effect exists, and this complicates efforts to substantiate strong recommendations in this field due to the lack of constancy with each of the above components amongst the studies included.

Diaz-Cambronero et al. have published extensively on this topic and appreciate the various elements involved, combining these measures to achieve an appropriate working space at lower pressures, leading to the development and testing of a “multifaceted individualized pneumoperitoneum strategy” [19, 35–37].

The role of neuromuscular blockade (NMB) has been observed across several clinical trials and studies. The NMB effect reduces the work of breathing and abdominal muscle contractions, which may improve the relationship between intra-abdominal pressure and volume. A review by Madsen et al. concluded that deep NMB in certain laparoscopic procedures (cholecystectomy, hysterectomy, nephrectomy and prostatectomy) may improve surgical conditions, however, good and excellent surgical conditions may be achievable even without NMB [32].

Interestingly, they observed that it was necessary to increase the intra-abdominal pressure in up to half of the patients regardless of the level of NMB [32]. Bruinjtes et al. reported significantly better laparoscopic surgical conditions and post-operative pain results when deep NMB was used compared with moderate NMB [30].

Within this meta-analysis, the significant reduction in pain associated with deep NMB occurred in the early postoperative period; and in conjunction with our review, it is unclear, whether this pain outcome is directly linked to the level of NMB itself or due to the lower intra-operative pressures obtained. Our review includes four trials reporting the varied use of NMB, which has limited our attempt at subgroup analysis, and this was the verbatim account of another recent review [29]. Consistency in reporting this parameter, along with the other influential measures, is required to establish the short and long-term implications of NMB on outcomes, and the optimum level of NMB that complements laparoscopic colorectal surgery.

Coelomic preservation plays an integral role in colorectal surgery. Peritoneal hypoxia and acidosis generated by CO_2_ promotes cytokine production and the malignant proliferation of cancers, particularly colorectal and gastric primaries [38–40]. Two of the reviewed studies collected data on the short-term post-operative inflammatory processes, with differing conclusions [17, 21]. Cai et al. reported a significant increase in cytokine production (serum only) on POD1 with no significant difference between high and low pressures [17]. Conversely, Albers et al. identified that low-pressure was associated with reduced surgical site hypoxia and inflammatory markers (serum and peritoneal biopsy), with a less-impaired early post-operative ex-vivo cytokine production capacity that was linked to improved pain scores and reduced infection rates [21].

Within our review, none of the studies reported on the long-term oncological outcomes in relation to the pneumoperitoneal pressures, possibly due to the short duration of post-operative follow-up devised or low retention. Future studies comparing low- and high-pressures in laparoscopic colorectal cancer surgery, are advised to report on the long-term oncological outcomes (survival rates, incidence of metastases and chemoradiotherapy responsiveness) to help consolidate an answer as to whether low-pressure CO_2_ insufflation reduces the risk of exponentiating tumorigenesis.

The role of CO_2_ emission in surgical procedures, particularly in optimising minimally invasive surgery has been extensively reported. The Royal College of Surgeons of England have regularly championed the ‘Green Surgery’ initiative through its reports and checklists [41, 42]. Furthermore an updated review by Robinson et al. found that the carbon footprint produced from surgical procedures ranges from 28 kg to half a ton [43]. Whilst this review has primarily addressed the clinical outcomes regarding insufflation pressures, we have a collective duty to acknowledge the important bio-economic implications of the global surgical use of CO_2_ on global warming and cost-implications [44]. With this in mind, and the concomitant understanding of the safety profile of low-pressure laparoscopy, we suggest that the environmental implications, of high insufflation pressures, should be considered in surgical decision making without compromising patient safety.

## Limitations

This review is not without its limitations. The lack of GRADE reporting of the individual trials limits our conclusion of the evidence and result certainty. Furthermore, this review conducted a thorough risk of bias assessment of all studies, reporting only a high risk of attrition bias. This is explained by the inconsistency and heterogeneity in outcome reporting across the included trials, limiting us from pooling pain scores, analgesia requirements, effects of NMB, and long-term outcomes. Although most of the included studies have defined low pressure as 7–8 mmHg, with high (standard) as 12–15 mmHg, the study by Cai et al. [17] measured low pressure as 10 mmHg, whilst Grieco et al. [20] compared pressures of 12 with 15 mmHg in patients undergoing transanal total mesorectal excision (TaTME). Methodological constancy is required to offer a true analysis of this multifaceted topic (pressure groups compared, surgical indications, and anaesthetic protocols used such as the NMB levels and baseline intra-operative and post-operative analgesia utilised). Furthermore, within the seven studies, cases were subject to the conversion of higher intraoperative pressures (for safety), and this dynamic adaptation, albeit necessary, adds to the attrition bias. This is the first review to provide high level evidence in the field of laparoscopic colorectal surgery and our findings form a strong foundation for ongoing and future studies, in the call for more consistency in methodology and reporting styles. We suggest the undertaking of a future review, once further well-designed clinical trials are available to pool and evaluate the true benefit of lower pressures in colorectal surgery on patient care.

## Conclusion

No statistically significant differences were observed in reported outcomes in patients undergoing laparoscopic colorectal surgery with either low or standard intraabdominal pressure. Lower pressures seem to provide a non-inferior, “greener”, safe and feasible laparoscopic alternative compared with standard high-pressure surgery.

Moreover, additional benefits may include improved post-operative pain and reduced analgesic requirement. However, future robust clinical trials with methodological consistency and reporting are needed to consolidate the potential benefits of low- over high-pressure in colorectal procedures.

## Data Availability

No datasets were generated or analysed during the current study.
